# Transfer Kernel Common Spatial Patterns for Motor Imagery Brain-Computer Interface Classification

**DOI:** 10.1155/2018/9871603

**Published:** 2018-03-18

**Authors:** Mengxi Dai, Dezhi Zheng, Shucong Liu, Pengju Zhang

**Affiliations:** ^1^Beijing Advanced Innovation Center for Big Data-Based Precision Medicine, Beihang University, Beijing 100191, China; ^2^Key Laboratory of Precision Opto-Mechatronics Technology, Ministry of Education, School of Instrumentation Science and Opto-Electronics Engineering, Beihang University, Beijing 100191, China

## Abstract

Motor-imagery-based brain-computer interfaces (BCIs) commonly use the common spatial pattern (CSP) as preprocessing step before classification. The CSP method is a supervised algorithm. Therefore a lot of time-consuming training data is needed to build the model. To address this issue, one promising approach is transfer learning, which generalizes a learning model can extract discriminative information from other subjects for target classification task. To this end, we propose a transfer kernel CSP (TKCSP) approach to learn a domain-invariant kernel by directly matching distributions of source subjects and target subjects. The dataset IVa of BCI Competition III is used to demonstrate the validity by our proposed methods. In the experiment, we compare the classification performance of the TKCSP against CSP, CSP for subject-to-subject transfer (CSP SJ-to-SJ), regularizing CSP (RCSP), stationary subspace CSP (ssCSP), multitask CSP (mtCSP), and the combined mtCSP and ssCSP (ss + mtCSP) method. The results indicate that the superior mean classification performance of TKCSP can achieve 81.14%, especially in case of source subjects with fewer number of training samples. Comprehensive experimental evidence on the dataset verifies the effectiveness and efficiency of the proposed TKCSP approach over several state-of-the-art methods.

## 1. Introduction

The brain-computer interface (BCI) offers a new pathway of communication between an external device and the brain through transforming metabolic or electrophysiological brain activities to control messages for devices and applications. The electroencephalogram (EEG) obtains time series data with multiple variants recorded at several sensors pressed on the scalp. It thereby presents electrical potentials under the induction of brain activities. These are used by noninvasive BCI systems to convert the mind or intention of a subject into a control message for certain device, such as a computer, a neuroprosthesis, or a wheelchair [1–4].

Currently, classification performance promotion of BCI systems based on the EEG has significant challenges. For one, it is necessary for a fresh subject to conduct a lengthy calibration session for sufficient training sample collection to establish classifiers and extractors of subject-specific features. The test session later employs the classifiers and extractors to classify the subjects brain signals. In a recent study on BCIs, it was shown to be very important to reduce training sessions on account of the time-consuming, tedious process of a calibration session. As a result, conducting a performance promotion using a scarce labeled set is more desirable compared with using a large one. Nevertheless, suitable methods must be identified to strengthen the performance. This is because a short calibration session means the availability of merely a few training samples for target users, which may result in overfitting or suboptimal feature classifiers or extractors.

To address the above problem, transfer learning is a promising approach [5, 6]. It applies data represented in various feature spaces or obtained from various distributions for compensating the insufficient labeled data. In the BCI field, transfer learning has attracted considerable attention because it enables the establishing of subject-independent spatial classifiers and/or filters, and it lowers calibration times. Some studies concentrated on feature representation transfer methods in EEG classification [7–11]. In this situation, we encode the knowledge that traverses domains into a fresh feature representation. Accordingly, precise classification performance is thereby expected in settings with a small sample.

**Figure 1:**
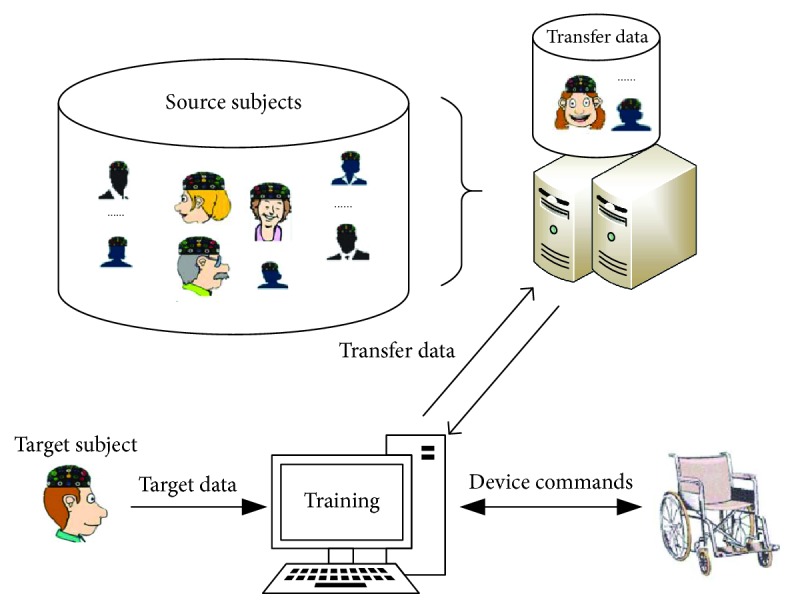
An illustration of subject transfer based BCI system.

A proposed schedule for practical applications of BCI systems based on subject transfer is presented in Figure 1 [12]. The datasets provided by the source subjects can be stored as a dataset group. Next, the BCI device can first acquire transfer data from the source subject groups when it is prepared to execute classification for the user. In this paper, we thus propose the transfer kernel common spatial patterns (TKCSP) method. The TKCSP computation is formulated by BCI as an optimization problem with multiple subjects, thereby incorporating data from other subjects to establish a common feature space.

## 2. Transfer Kernel Common Spatial Patterns

This section mentioned a new feature extraction method, TKCSP, which combines two previous approaches, kernel common spatial patterns (KCSP) [13] and transfer kernel learning (TKL) [6]. KCSP is an extraction approach for motor imagery, and TKL is a promising transfer learning method. In Sections 2.1 and 2.2, we describe the KCSP algorithm and the TKL algorithm, respectively. In Section 2.3, we would propose the TKCSP algorithm in combination with the above two algorithms.

### 2.1. Kernel Common Spatial Patterns

The KCSP algorithm based on CSP is used to find the components with the largest energy difference between the two experimental conditions [13–15]. Its basic idea is to find the optimal spatial filter to maximize the component energy under two sets of experimental conditions after the spatial filtering.

The first step is to calculate the covariance between the two signals. Consider *E*_*i*_ as an *M* × *T* matrix representing the *i*th trial of EEG signals, wherein *M* represents the channel amount and *T* represents the points of time. The class-specific spatial covariance matrix can hence be acquired by the steps below.

(1) $$\begin{array}{c} R_{i}=\frac{K\left(E_{i}E_{i}^{T}\right)}{\text{trace}\left(K\left(E_{i}E_{i}^{T}\right)\right)}, \end{array}$$

where *i* represents the class label, *K*(*E*_*i*_*E*_*i*_^*T*^) = 〈*φ*(*E*_*i*_), *φ*(*E*_*i*_)〉 represents the kernel function, and 〈 〉 denotes the inner product. Thus we can replace the computation of the aggregate spatial covariance matrix with *R*_*c*_ = *R*_1_ + *R*_2_.

Additionally, we can factor *R*_*c*_ to be *R*_*c*_ = *U*_0_Λ_*c*_*U*_0_^*T*^, where *U*_0_ ∈ **R**^*n*×*n*^ represents a matrix with eigenvectors in a row, while Λ_*c*_ represents the diagonal matrix of eigenvalues classified in declining order.

The variances can be equalized by using a whitening transmission *P* within space that the eigenvectors span in *U*_0_ such that *P* equals

(2) $$\begin{array}{c} P=\lambda^{1/2}U_{0}^{T}. \end{array}$$

Thirdly, the whitening matrix *P* can be used to transform *R*_1_ and *R*_2_ into *S*_1_ and *S*_2_ as

(3) S1=PR1PT,S2=PR2PT.

*S*_1_ and *S*_2_ have the same eigenvectors, that is, if

(4) S1=Bλ1BT,S2=Bλ2BT,λ1+λ2=I,

where *I* represents the identity matrix. At this point, the sum is always one for these two corresponding eigenvalues. Hence, the eigenvectors having the smallest eigenvalues for *S*_1_ have the largest eigenvalues for *S*_2_ and vice versa. This property enables eigenvector *B* to sort these two classes.

Finally, owing to *W* = (*B*^*T*^*P*)^*T*^ as the common spatial filters, the common spatial patterns are columns of *W*^−1^, which can be regarded as the source distribution vectors for time-invariant EEG.  Algorithm 1 shows the summary of a complete KCSP procedure.

### 2.2. Transfer Kernel Learning

TKL can directly match the source distribution and target distribution to learn a domain-invariant kernel space, using the knowledge of the source domain to help complete the learning tasks in the target domain. This section begins with definitions of terminology used, and Notations section presents a summary of commonly used notations.

**Definition 1 .**

A domain *D* includes a *d*-dimensional feature space *ℱ* as well as a marginal probability distribution *P*(*x*); that is, *𝒟* = {*ℱ*, *P*(*x*)}, *x* ∈ *ℱ*.

In general, if two domains *Z* and *X* have different marginal distributions or feature spaces, they will have difference; that is, *ℱ*_*Z*_ ≠ *ℱ*_*X*_∨*P*(*z*) ≠ *P*(*x*).

**Definition 2 .**

Given domain *𝒟*, a classifier *f*(*x*) and a cardinality label set *𝒴* compose a task *𝒯*; that is, *𝒯* = {*𝒴*, *f*(*x*)}, in which *y* ∈ *𝒴*, and the interpretation of *f*(*x*) = *P*(*y*∣*x*) can be conditioned probability distribution.

In general, if two tasks *𝒯*_*Z*_ and *𝒯*_*X*_ have different conditioned distributions or label spaces, they will have a difference; that is, *𝒴*_*Z*_ ≠ *𝒴*_*X*_∨*P*(*y*∣*z*) ≠ *P*(*y*∣*x*).

**Problem 3 (transfer kernel learning).**

Given an unlabeled target domain *X* = {*x*_1_,…, *x*_*n*_} and a labeled source domain *Z* = {(*z*_1_, *y*_1_),…, (*z*_*m*_, *y*_*m*_)} with *ℱ*_*Z*_ = *ℱ*_*X*_, *𝒴*_*Z*_ = *𝒴*_*X*_, *P*(*z*) ≠ *P*(*x*), and *P*(*y*∣*z*) ≠ *P*(*y*∣*x*), a kernel *k*(*z*, *x*) = 〈*ϕ*(*z*), *ϕ*(*x*)〉 with an invariable domain is learned so that *P*(*ϕ*(*z*))≃*P*(*ϕ*(*x*)). Suppose *P*(*y*∣*ϕ*(*z*))≃*P*(*y*∣*ϕ*(*x*)), then a kernel machine targeting *Z* can effectively generalize *X*.

Firstly, calculate the target kernel function, the source kernel function, and the cross-kernel function. Assume an input kernel function *k* is given to us, for example, Laplacian kernel *k*(*z*, *x*) = *e*^*γ*|*z* − *x*|^ or Gaussian kernel *k*(*z*, *x*) = *e*^*γ*‖*z* − *x*‖^2^^, then the target kernel *K*_*X*_, the source kernel *K*_*Z*_, and the cross-domain kernel *K*_*ZX*_ can be computed. A domain-invariant kernel ${\bar{K}}_{Z\cup X}$ can be learned by utilizing these three kernels. Under this challenging situation, the sufficient matching of marginal distributions plays an indispensable role in efficient learning of the domain transfer.

**Figure 2:**
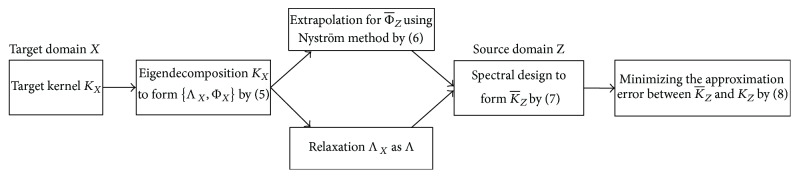
Complete procedure of transfer kernel learning.

To require two datasets (for example, target data *X* and source data *Z*) to conform to similar distributions of the feature space, that is, *P*(*ϕ*(*z*))≃*P*(*ϕ*(*x*)), requiring them to have similar kernel matrices is sufficient, that is, *K*_*Z*_≃*K*_*X*_ [16]. Nevertheless, kernel matrices depend on data and the direct evaluation of closeness between varied kernels is improbable because of the varying dimensions; that is, *K*_*Z*_ ∈ *R*^*m*×*m*^, *K*_*X*_ ∈ *R*^*n*×*n*^ [17]. To solve this issue, the Nystr$\ddot{\text{o}}$m kernel approximation idea is adopted for the generation of an extrapolated source kernel ${\bar{K}}_{Z}\in R^{m\times m}$ by an eigensystem of target kernel *K*_*X*_. Next, ${\bar{K}}_{Z}$ can arise to kernel *K*_*Z*_ as the ground truth source and can be comparable to a spectral kernel design.  Figure 2 shows the whole learning procedure.

Secondly, Nystr$\ddot{\text{o}}$m kernel approximation is adopted to execute eigensystem extrapolation [16]. To this end, standard eigendecomposition is adopted on the target kernel *K*_*X*_

(5) $$\begin{array}{c} K_{X}\Phi_{X}=\Phi_{X}\Lambda_{X}, \end{array}$$

which provides the eigensystem {Λ_*X*_, Φ_*X*_} of target kernel *K*_*X*_.

Thirdly, we assess the eigensystem on source data *Z* by utilizing the Nystr$\ddot{\text{o}}$m approximation theorem. The derivation of the eigenvectors ${\bar{\Phi }}_{Z}$ for extrapolated source kernel ${\bar{K}}_{Z}$ is

(6) $$\begin{array}{c} {\bar{\Phi }}_{Z}≃K_{ZX}\Phi_{X}\Lambda_{X}^{-1}, \end{array}$$

where *K*_*ZX*_ ∈ **R**^*m*×*n*^ is the cross-domain kernel matrix between *Z* and *X*, assessed by kernel function *k*.

The initial Nyström method directly utilizes target eigenvalues Λ_*X*_ and extrapolated source eigenvectors ${\bar{\Phi }}_{Z}$ to make approximation for the source kernel *K*_*Z*_. In essence, the distribution difference across domains is embodied by the Nystr$\ddot{\text{o}}$m approximation error; that is, error is close to 0 if and only if *P*(*z*)≃*P*(*x*). An invariant kernel extrapolated to varied domains will be achieved if an extrapolated kernel can be found for realizing a minimized $\text{Nystr}\ddot{\text{o}}\text{m}$ approximation error, thereby facilitating a more efficient cross-domain generalization.

The spectral kernel design idea is adopted to establish a new kernel matrix from extrapolated eigensystem to reduce the Nyström approximation error [18]. The key construction of target kernel *K*_*X*_ can thus be preserved by the kernel matrix generated via extrapolated eigensystem Φ_*Z*_; however, the flexibility of the reshaping could be retained to keep the distribution difference minimized.

Fourthly, eigenspectrum Λ_*X*_ can be relaxed in the primary $\text{Nystr}\ddot{\text{o}}\text{m}$ approach to be parameters Λ that can be learned resulting in a kernel family extrapolated from the target eigensystem yet assessed on the source data. The extrapolated source kernel ${\bar{K}}_{Z}$ is obtained as follows:

(7) $$\begin{array}{c} {\bar{K}}_{Z}={\bar{\Phi }}_{Z}\Lambda {\bar{\Phi }}_{Z}^{T}. \end{array}$$

The critical structures of the target domain can be preserved by this kernel family, that is, eigenvectors ${\bar{\Phi }}_{Z}$. Moreover, the free eigenspectrum Λ remains undetermined. Unlike a conventional spectral kernel design that learns the parameters through Λ trained on the spectral kernel towards a previous kernel calculated in the same domain, kernel matching can be performed across domains.

Fifthly, we strive to minimize the approximation error between the ground truth source kernel *K*_*Z*_ and the extrapolated source kernel ${\bar{K}}_{Z}$ for explicitly minimizing the distribution difference herein by utilizing the squared loss

(8) minΛ⁡  K¯Z−KZF2=Φ¯ZΛΦZT−KZF2 λi≥ζλi+1,i=1,…,n−1 λi≥0,i=1,…,n,

where Λ = diag⁡(*λ*_1_,…, *λ*_*n*_) belongs to the *n* nonnegative eigenspectrum parameters, while *ζ* ≥ 1 belongs to the eigenspectrum damping factor [19].

The marginal distributions of multiple source domains can be matched with the target domain using the generalized transfer kernel learning (TKL) approach. This approach can be conducted by the source-specific eigenspectrum Λ learning for every source domain separately in the initial place. Secondly, existing learning algorithms of multiple sources are used to implement consensus forecasting for the target domain on the basis of predicting multiple source domains [20, 21].

Sixthly, the standard quadratic programming possessing (QP) linear constraints are used herein to show the solution of the TKL optimization problem (8). Here, *n* eigenspectrum parameter is denoted as ***λ*** = (*λ*_1_,…, *λ*_*n*_); that is, Λ = diag⁡(***λ***). Equation (8) is reformulated in the matrix form by linear algebra

(9) minλ⁡  λTQλ−2γTλ Cλ≥0 λ≥0.

The following are the respective definitions of QP coefficient matrices *Q*, *γ* and constraint matrix

(10) Q=ΦZTΦZ⊙ΦZTΦZγ=diag⁡ΦZTKZΦZC=I−ζI¯,

where *ζ* ≥ 1 represents the eigenspectrum damping factor, which is also the only tunable parameter within TKL. Additionally, *I* ∈ **R**^*n*×*n*^ denotes the identity matrix, and $\bar{I}\in R^{n\times n}$ represents the first diagonal matrix with the nonvanishing elements.

Finally, constructing the domain-invariant kernel ${\bar{K}}_{A}$ on the target and source data *A* = *Z* ∪ *X* is straightforward with the learned optimal eigenspectrum parameters Λ. According to spectral kernel design, we can generate ${\bar{K}}_{A}$ from the eigensystem $\left\{\Lambda ,{\bar{\Phi }}_{A}\right\}$ invariant to domain

(11) $$\begin{array}{c} {\bar{K}}_{A}=\left[\begin{array}{cc} {\bar{\Phi }}_{Z}\Lambda {\bar{\Phi }}_{Z}^{T} & {\bar{\Phi }}_{Z}\Lambda \Phi_{X}^{T}\\ {\bar{\Phi }}_{Z}\Lambda {\bar{\Phi }}_{X}^{T} & {\bar{\Phi }}_{X}\Lambda \Phi_{X}^{T} \end{array}\right]={\bar{\Phi }}_{A}\Lambda {\bar{\Phi }}_{A}^{T}, \end{array}$$

where ${\bar{\Phi }}_{A}≜[{\bar{\Phi }}_{Z};\Phi_{X}]$ belongs to extrapolated eigenvectors on all data *A*. We can directly feed the kernel ${\bar{K}}_{A}$ invariant to the domain to normal kernel machines, for example, KCSP, for facilitating the cross-domain generalization and prediction.  Algorithm 2 shows the summary of a complete procedure.

### 2.3. Transfer Kernel CSP

When transfer kernel ${\bar{K}}_{A}$ replaces kernel *K*(*E*_*i*_*E*_*i*_^*T*^) in (1), we can build the TKCSP. For all methods based on the kernel, linear kernel is adopted by us; that is, *K*(*x*_*i*_, *x*_*j*_) = *x*_*i*_^*T*^*x*_*j*_. Then *R*_*i*_ = *K*_*A*_/trace(*K*_*A*_) can be used to estimate the spatial covariance.  Algorithm 3 presents a summary of a complete TKCSP procedure.

We can compute the filtration of a trial *E*_*j*_ by *W* = (*B*^*T*^*P*)^*T*^ as the projection matrix [14]:

(12) $$\begin{array}{c} Z_{i}=W\times E_{i}. \end{array}$$

Decomposing the EEG based on (6) can be used to obtain the features utilized for classification. For every imagined movement direction, the classifier construction employs the variances owned by merely a small amount of *m* signals that are the fittest for discrimination. The signals *Z*_*p*_  (*p* = 1 ⋯ 2*m*) maximizing the variance difference of motor imagery EEG on the left versus the right belong to those associated with the largest eigenvalues *λ*_1_ and *λ*_2_. These signals are blank in the last and first rows in *Z* because of the computation of *W*

(13) $$\begin{array}{c} f_{p}=\frac{\mathrm{var}\left(Z_{p}\right)}{\sum_{i=1}^{2m}\mathrm{var}\left(Z_{i}\right)}. \end{array}$$

The linear classifier can be calculated by using the feature vectors *f*_*p*_ of right and left trials. The log-transformation contributes to approximating the standard data distribution.

## 3. Experiments

### 3.1. Data Preparation

In this study, we employed the IVa dataset from BCI Competition III [22]. The dataset includes EEG data containing a classification task of motor imagery with two levels: (1) imagery movement of the right hand (denoted by R) and (2) imagery movement of the right foot (denoted by F). We employed 118 electrodes to measure EEG signals in every trial from five different subjects, and each subject involved the performance of 280 trials.  Table 1 presents a summary of the data descriptions, in which the number of subjects av, aw, ay of training samples is fewer than those of the test samples.

Each trial was considered an *M* × *T* matrix *E*_*i*_, in which *M* represents the electrode amount and the time point amount sampled. EEG signals measured were band-pass decomposed (8–30 Hz). SVM (Support Vector Machine) involving linear kernel was utilized as the classifier. The proportion of the number of samples properly classified to the aggregate number of used samples in this test was employed to evaluate the classification precision.

Our establishment of a dataset (containing the target domain and source domain) for cross-domain classification is described as follows. The dataset of each subject could become the target domain (ay, aw, av, al, aa), while the dataset of other subjects could become the source domains. This strategy of dataset construction ensured the relevance between domains of unlabeled and labeled data, as they were located in the same top-level categories. Accordingly, *C*_4_^1^ + *C*_4_^2^ + *C*_4_^3^ + *C*_4_^4^ = 15 datasets of the source domains were generated for each target domain. It was possible to generate five dataset groups, including 5 × 15 = 75 datasets.

### 3.2. Experimental Results

In this section, TKCSP and six competitive methods are evaluated based on classification accuracy [8, 11, 23]. We established five dataset groups from the dataset described above. Each dataset group includes four source subjects in source domains and one target subject as target domain. If one subject is the target domain, it will no longer appear in the source domains, so that each target domain corresponds to 15 different source domains. The first column of Table 2 shows the different source domains, and the second column to the sixth column of Table 2 show the classification accuracy of each target domain in its source domains, respectively. Among them, the highest classification accuracy of target domain aa was 68.10% and the corresponding source domain was al + aw; the highest classification accuracy of target domain al was 93.88% and the corresponding source domain was aw; the highest classification accuracy of target domain av was 68.47% and the corresponding source domain was al + ay; the highest classification accuracy of target domain aw is 68.10% and the corresponding source domain is al; the highest classification accuracy of target domain ay is 68.10% and the corresponding source domain is aa + al.

**Figure 3:**
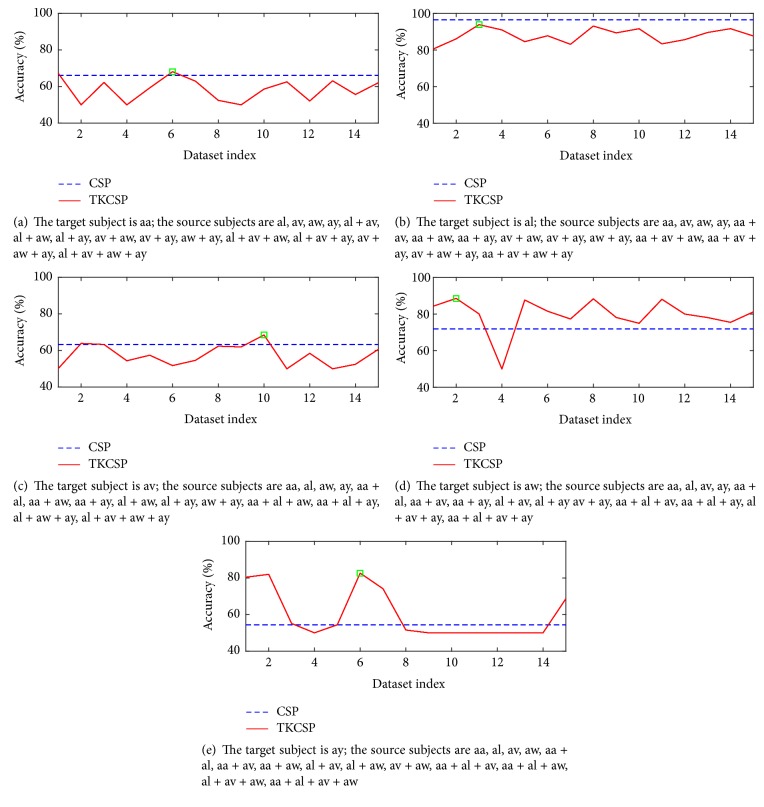
Classification accuracy of TKCSP and CSP on the dataset.

To be complete, we detail the results of TKCSP method and CSP approach on all of 5 dataset groups in Figures 3(a), 3(b), 3(c), 3(d), and 3(e), where each figure presents the results of each group. In Figure 3, the blue dashed line indicates the classification accuracy of CSP algorithm, the red solid line indicates the classification accuracy of TKCSP algorithm in different source domains, and the green square indicates the best classification accuracy of TKCSP. The horizontal axis of green square is corresponding to the optimal source domain. The results show that the classification accuracy of TKCSP method is better than that of CSP algorithm.

**Figure 4:**
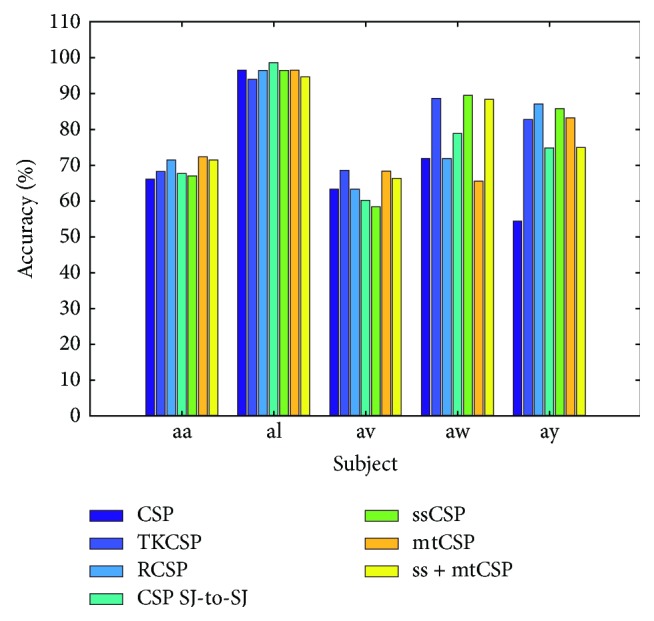
Comparison of classification accuracies for TKCSP and 6 competitive methods.

Table 3 lists the classification (recognition) precisions of five comparison approaches and TKCSP on dataset IVa.  Figure 4 visually depicts the results for improved accessibility. The performance achieved by TKCSP is significantly better than those achieved by the five comparison approaches. Several observations can be made from these results.

Firstly, TKCSP achieves classification precision on the aw and aa datasets as 90.58% and 68.47%, respectively. These are higher than those of the five comparison approaches. Moreover, TKCSP achieves an average classification precision on these datasets as 81.14%, providing a significant performance improvement of 1.97% over ss + mtCSP, the best competitive approach. It is strongly verified by the consistent performance improvements on these datasets that TKCSP can successfully establish powerful domain kernels for classification of cross-domain motor imagery.

Then, a composite covariance matrix as a weighted total of covariance matrices, including subjects resulting in a composite CSP, is determined by CSP for subject-to-subject transfer (CSP SJ-to-SJ). This approach thus achieves an average classification precision of 75.97%.

Thirdly, regularizing CSP (RCSP) is intended to regularize the covariance matrix to the mean covariance matrix of other subjects for improving its estimation performance. Such regularization is particularly promising in settings with small samples. Furthermore, this approach achieves an average classification precision of 77.98%.

Finally, the stationary subspace CSP (ssCSP) focuses on the nonstationarity issue while multitask CSP (mtCSP) focuses on the estimation issue. The combined mtCSP and ssCSP (ss + mtCSP) method employs both approaches. That is, the nonstationary subspace acquired by ssCSP is firstly projected, and then the spatial filters are computed with mtCSP by regularization parameters acquired when it is applied to the initial data. The three above approaches achieve an average classification precision of 78.99%, 76.81%, and 79.17%, respectively.

In particular, TKCSP can assess the various cluster structures and naturally matches them between multiple domains. This procedure is achieved by TKCSP through the matching between the source domain kernel and kernel extrapolated from the target domain, while simultaneously increasing (declining) the domain-invariant (domain independent) eigenspectrum. The superior performance of TKCSP can be explained by this advantage.

## 4. Conclusion

In this paper, we proposed the TKCSP method to lower the training trial amount and improve the performance via learning a domain-independent kernel. To this end, direct matching of distributions between target subjects and source subjects within the kernel space is conducted. TKCSP and six competitive approaches were evaluated on EEG datasets provided by BCI Competition III. The results showed that the performance of the best approach, RCSP, was better than that of CSP by nearly 1.97% in terms of the mean classification precision. The results also revealed that RCSP can perform effective subject-to-subject transfer. Therefore, the behaviors matched with knowledge of neurophysiology could be classified by the TKCSP approach.

## Figures and Tables

**Algorithm 1:**
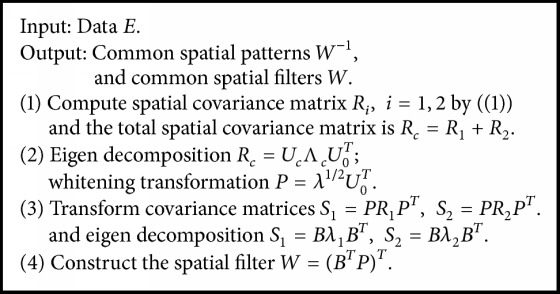
Kernel common spatial pattern algorithms.

**Algorithm 2:**
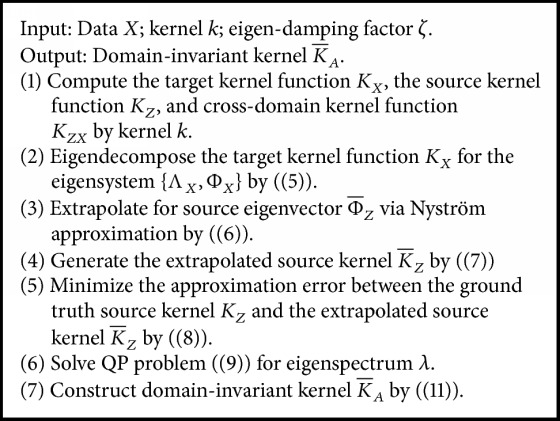
TKL algorithm.

**Algorithm 3:**
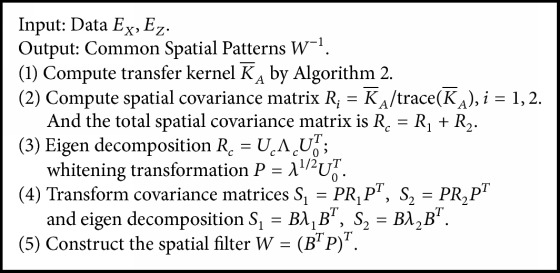
Transfer kernel common spatial pattern algorithm.

**Table 1:** Data description for dataset IVa in BCI Competition III.

Subject	aa	al	av	aw	ay
Number of training samples	168	224	84	56	28
Number of test samples	112	56	196	224	252

**Table 2:** Classification accuracy of TKCSP on the dataset.

Source	Target				
	aa (%)	al (%)	av (%)	aw (%)	ay (%)
aa	-	80.58	50.17	84.33	80.41
al	67.20	-	63.87	**90.58**	81.98
av	50.00	86.11	-	80.10	55.13
aw	62.23	**93.88**	54.39	-	50.00
ay	50.00	91.00	65.67	50.00	-
aa + al	-	-	57.43	87.69	**84.65**
aa + av	-	84.58	-	81.56	74.11
aa + aw	-	87.80	51.76	-	51.53
aa + ay	-	84.58	-	81.56	74.11
al + av	59.27	-	-	88.35	50.00
al + aw	**68.10**	-	61.96	-	50.00
al + ay	62.92	-	**68.47**	78.16	-
av + aw	52.44	93.11	-	-	50.00
av + ay	45.30	89.38	-	74.98	-
aw + ay	58.60	91.65	61.25	-	-
aa + al + av	-	-	-	88.10	73.25
aa + al + aw	-	-	50.00	-	58.60
aa + al + ay	-	-	58.47	80.01	-
aa + av + aw	-	83.37	-	-	50.00
aa + av + ay	-	85.68	-	78.10	-
aa + aw + ay	-	89.59	50.00	-	-
al + av + aw	62.56	-	-	-	50.00
al + av + ay	52.10	-	-	75.48	-
al + aw + ay	63.13	-	52.47	-	-
av + aw + ay	55.65	91.67	-	-	-
al + av + aw + ay	62.07	-	-	-	-
aa + av + aw + ay	-	87.68	-	-	-
aa + al + aw + ay	-	-	60.63	-	-
aa + al + av + ay	-	-	-	81.20	-
aa + al + av + aw	-	-	-	-	53.19

**Table 3:** Comparison of classification accuracy for TKCSP and 6 competitive methods.

Subject	aa	al	av	aw	ay	Mean
	(%)	(%)	(%)	(%)	(%)	(%)
CSP	66.07	96.43	63.30	71.88	54.40	70.42
RCSP	71.43	96.43	63.30	71.88	86.90	77.98
CSP SJ-to-SJ	67.76	98.41	60.20	78.72	74.78	75.97
ssCSP	67.00	94.62	58.26	89.35	85.71	78.99
mtCSP	72.33	94.62	68.39	65.57	83.14	76.81
ss + mtCSP	71.43	94.63	66.32	88.40	74.93	79.17
TKCSP	68.10	93.88	68.47	90.58	84.65	81.14

## Notations

*Z*, *X*: Source/target domain
*m*, *n*: Source/target examples
*d*, *c*: Features/classes
*γ*, *ζ*: Eigenvectors/damping factor
**X**: Input data matrix
**K**: Kernel matrix
Φ: Eigenvector matrix
Λ: Eigenvalue matrix.
